# Ouabain Promotes Gap Junctional Intercellular Communication in Cancer Cells

**DOI:** 10.3390/ijms22010358

**Published:** 2020-12-31

**Authors:** Mauricio Serrano-Rubi, Lidia Jimenez, Jacqueline Martinez-Rendon, Marcelino Cereijido, Arturo Ponce

**Affiliations:** Department of Physiology, Biophysics and Neurosciences, CINVESTAV-IPN, Mexico City C.P. 07360, Mexico; mauricio.serrano@cinvestav.mx (M.S.-R.); lidia.jimenez@cinvestav.mx (L.J.); jamare@fisio.cinvestav.mx (J.M.-R.); cereijido@fisio.cinvestav.mx (M.C.)

**Keywords:** gap junctions, ouabain, cancer

## Abstract

Gap junctions are molecular structures that allow communication between neighboring cells. It has been shown that gap junctional intercellular communication (GJIC) is notoriously reduced in cancer cells compared to their normal counterparts. Ouabain, a plant derived substance, widely known for its therapeutic properties on the heart, has been shown to play a role in several types of cancer, although its mechanism of action is not yet fully understood. Since we have previously shown that ouabain enhances GJIC in epithelial cells (MDCK), here we probed whether ouabain affects GJIC in a variety of cancer cell lines, including cervico-uterine (CasKi, SiHa and Hela), breast (MDA-MB-321 and MCF7), lung (A549), colon (SW480) and pancreas (HPAF-II). For this purpose, we conducted dye transfer assays to measure and compare GJIC in monolayers of cells with and without treatment with ouabain (0.1, 1, 10, 50 and 500 nM). We found that ouabain induces a statistically significant enhancement of GJIC in all of these cancer cell lines, albeit with distinct sensitivity. Additionally, we show that synthesis of new nucleotides or protein subunits is not required, and that Csrc, ErK1/2 and ROCK-Rho mediate the signaling mechanisms. These results may contribute to explaining how ouabain influences cancer.

## 1. Introduction

Ouabain is a compound of remarkable interest that—because of its chemical properties—has been useful to humans for a long time, formerly for hunting and war purposes and later as a medicine to treat hypotension and cardiac arrhythmias. Ouabain is a cardiac glycoside, produced as a secondary metabolite by plant species *Acokanthera schimperi* and *Strophanthus gratus*, both native to eastern Africa [1]. It is known that ouabain induces a positive inotropic effect on the heart because it inhibits Na/K ATPase, leading to an increase in intracellular sodium, which in turn reduces the activity of the sodium-calcium exchanger (NCX), which elicits an increased intracellular calcium concentration, that enhances the cardiac output.

Ouabain has been used as a cardiotonic in the past but, due to its narrow therapeutic index, it has now been discontinued [2]. However, two factors have again aroused interest in this substance: firstly, the fact that ouabain has been found to be endogenously expressed in some mammal species, including humans, has led to it being considered as a hormone [3,4,5,6] and secondly, that Na/K ATPase has now been attributed a new role, as the receptor that transduces the binding of ouabain to trigger one or more signaling cascades, leading to genetic and functional modifications of various cell processes [7,8].

In addition to the well-known effects of ouabain on the cardiovascular system and blood pressure control, compelling findings have revealed new roles in fundamental cellular aspects, such as proliferation, apoptosis, cell adhesion, differentiation and migration [9,10]. These findings have led to ouabain being considered as a promising novel therapeutic agent to fight cancer [11,12].

Gap junctions are molecular structures consisting of clusters of channels co-expressed between two neighboring cells, allowing them to exchange ions and small molecules. A gap junction channel consists of two hemichannels (connexons), one contributed by each cell. In turn, each connexon consists of six connexin subunits [13,14,15]. From the pioneering work of Loewenstein and Kanno, which describes that cancerous liver cells have reduced gap junctional intercellular communication (GJIC) [16,17], experimental evidence has accumulated demonstrating a relationship between GJIC and the expression of connexins and cancer-related properties. On the one hand, it has been described that decreased connexin expression and loss of GJIC is, in many instances associated with cancer onset and progression [18,19] and conversely, that re-expression of connexins in cancer cells normalizes cell growth control and reduces tumor growth [20]; however on the other hand, several reports show that re-expression of connexins and intense GJIC are associated with a high invasive potential and metastatic capability of cancer cells [21,22,23].

We showed that ouabain, in a nanomolar range of concentration, influences several properties of epithelial cells, including tight junctions, adherens junctions and ciliogenesis [24,25,26]. Additionally, by dye transfer assays, as well as capacitance measurements, we have shown that it enhances GJIC within minutes of treatment, with a peak response after one hour [27]. We have also shown that: (1) connexins 32 and 43 are involved in this response; (2) no synthesis of new connexin subunits is required within one hour of treatment and instead the process relies on a relocation of previously synthesized connexin subunits; and (3) that Na/K ATPase is the primary receptor that mediates a signaling cascade, involving c-Src and ERK1/2 [28]. These results prompted us to test whether ouabain influences GJIC in cancer derived cell lines. For this purpose, we selected several cancerous cell lines derived from distinct origins, including cervico-uterine (CaSki, SiHa and HeLa), breast (MCF7, MDA-MB-231), lung (A549), colon (SW480) and pancreas (HPAF-II). In each of them, we made dye transfer assays, to measure and compare GJIC at several ouabain concentrations in the nanomolar range.

## 2. Results

**Figure 1:**
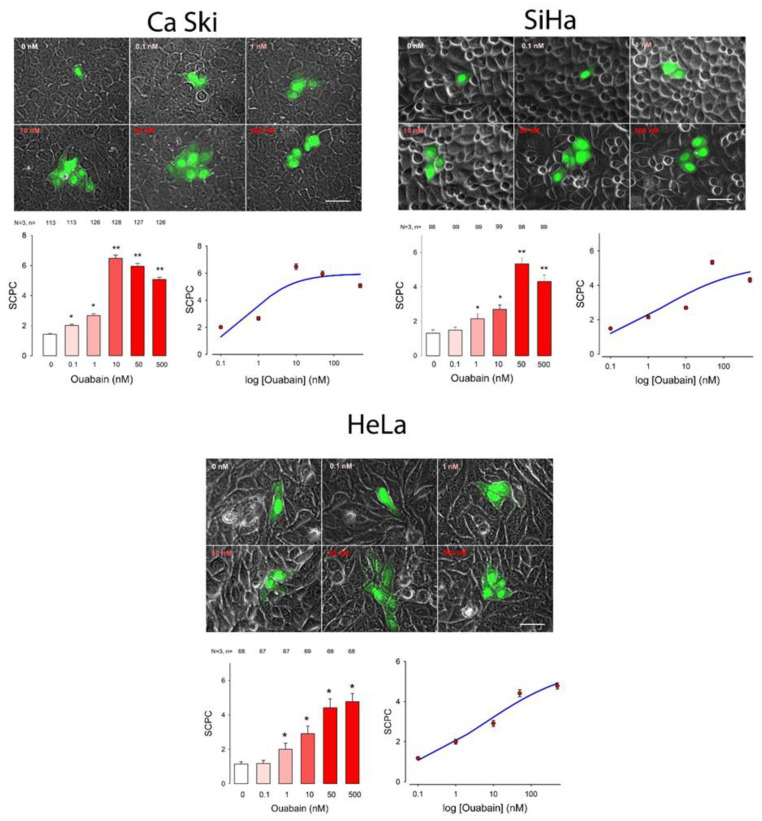
Effect of ouabain on gap junctional intercellular communication (GJIC) of cervico-uterine cancer cell lines (CasKi, SiHa and Hela). Each set shows, in the top part: representative images comparing the number of stained cell per cluster (SCPC) after one of them had been injected with Lucifer Yellow, from monolayers that were either untreated (0 nM) or treated for one hour with ouabain in the concentration indicated (nM). In the bottom part: (**Left**) Histogram showing the average (± SE) SCPC in monolayers of confluent cells that were treated with ouabain, for one hour, in the concentrations indicated at the bottom of the bars. At the top of each bar the number of repeats after three independent trials is shown. Asterisks indicate a statistically significant difference compared to the control group, (Dunn’s method), * indicates *p* < 0.05, ** indicates *p* < 0.001. Scale bar length = 100 µM. (**Right**) A semi-log plot shows mSCPC ± SE (red) and the curve (blue) resulting after fitting data to a logistic equation.

**Figure 2:**
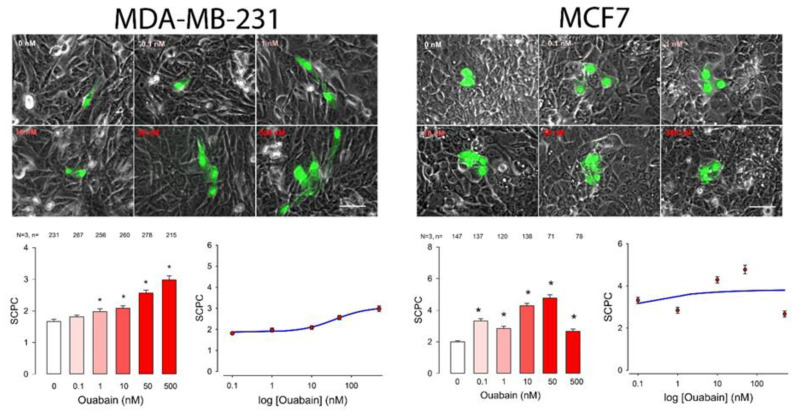
Effect of ouabain on GJIC of breast cancer cell lines (MDA-MB-231 and MCF7). Each set shows, in the top part: representative images comparing the number of stained cell per cluster (SCPC) after one of them had been injected with Lucifer Yellow in monolayers that were either untreated (0 nM) or treated for one hour with ouabain in the concentration indicated (nM). In the bottom part: (**Left**) Histogram showing mSCPC (± SE) in monolayers of confluent cells that were treated with ouabain, for one hour, in the concentrations indicated at the bottom of the bars. At the top of each bar the number of repeats after three independent trials is shown. Asterisks indicate a statistically significant difference compared to the control group, (Dunn’s method), * indicates *p* = < 0.05. Scale bar length = 100 µM. (**Right**) A semi-log plot shows, the average data and the curve (blue) resulting after fitting data to a logistic equation.

**Figure 3:**
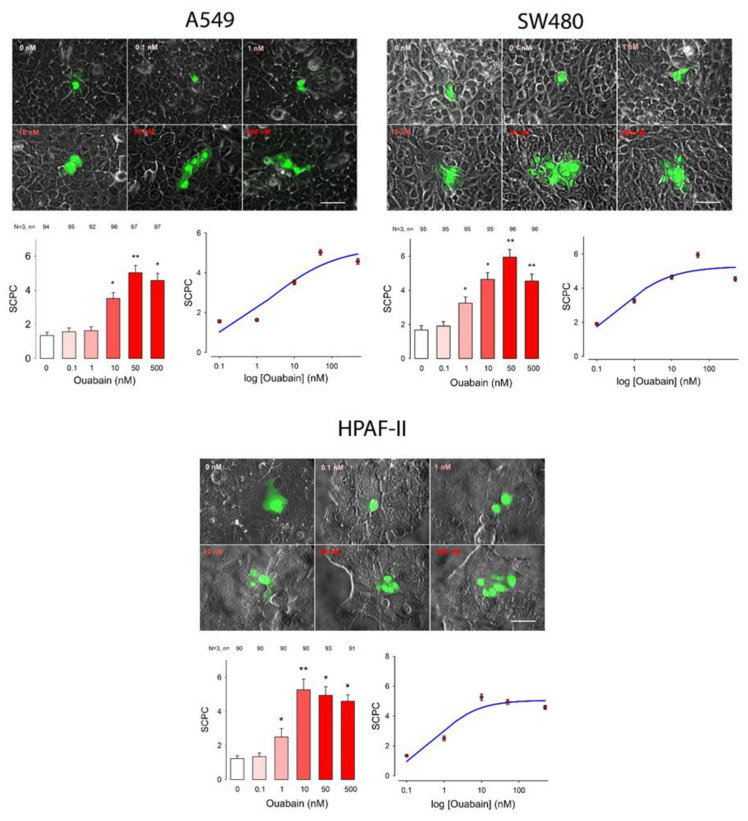
Effect of ouabain on GJIC in lung (A549), colon (SW480) and pancreas (Hpaf-II) cell lines. Each set shows, in the top part: representative images comparing the number of stained cell per cluster (SCPC) after one of them had been injected with Lucifer Yellow in monolayers that were either untreated (0 nM) or treated for one hour with ouabain in the concentration indicated (nM). In the bottom part: (**Left**) Histogram showing the mSCPC (± SE) in monolayers of confluent cells that were treated with ouabain, for one hour, in the concentrations indicated at the bottom of the bars. At the top of each bar the number of repeats after three independent trials is shown. Asterisks indicate a statistically significant difference compared to the control group, (Dunn’s method), * indicates *p* = < 0.05, ** indicates *p* = < 0.005). Scale bar length = 100 µM. (**Right**) A semi-log plot shows, the average data and the curve (blue) resulting after fitting data to a logistic equation.

To determine whether ouabain influences GJIC we made dye transfer assays in the different cancer cell lines. The number of stained cells per case (SCPC) was an estimate of GJIC. First, we made an ANOVA analysis (Kruskal—Wallis), to probe the hypothesis that ouabain induces a significant change on GJIC. Next, we compared the results of each of the ouabain concentrations with the control (Dunn test) to determine which concentrations produced a statistically significant difference. In addition to this, we fitted the data, by nonlinear regression, to a dose-response curve (Hill Equation) to obtain the parametric values (EC_50_ and SCPC_max_). This experimental approach and analysis were performed separately on each of the cell lines described above. The results, as well as the analysis, are shown in Figure 1, Figure 2 and Figure 3 and summarized in Table 1 and Table 2. Figure 1, Figure 2 and Figure 3 consist of several sets, one per each cell line. In turn each set shows, in the upper part, a series of representative images of clusters of stained cells, one for each concentration of ouabain, including the control. The bottom left part presents a bar chart comparing the mean value of SCPC (mSCPC) at each ouabain concentration with the control group. The bottom left part shows the same data plus the fitted curve. The results of the statistical analyses are summarized in Table 1 and the parametric values obtained after fitting of data to Hill equation, are summarized in Table 2. Figure 1 shows the results of cervico-uterine cancer cell lines (CaSki, SiHa and HeLa), Figure 2 shows the results of breast cancer cell lines (MDA-MB-231 and MCF7) and Figure 3 shows the results of lung (A549), colon (SW480) and pancreas (Hpaf-II) cancer cell lines. As figures and Table 1 show, ouabain induced a statistically significant increase of GJIC in all the cell lines tested. This conclusion is demonstrated by the fact that the Kruskal-Wallis test was significant (*p* < 0.001) in all of them. Multiple comparison tests (Duncan method) indicate that ouabain induced a significant change from 1 nM in almost all cell lines, except MCF7 in which a significant change was observed from 0.1 nM and A549 where it occurred from 10 nM. As the bar charts show, the profile of the dose-response relationship had similar characteristics in the different cell lines. Under control conditions, mSCPC was between one and two, confirming scarce GJIC. In most of the cell lines, mSCPC increased with the concentration of ouabain to a maximum, and then decreased slightly. In SiHa, MCF7, A549, SW480 cell lines, the maximum was reached at 50 nM, while in CaSki and Hpaf-II it was at 10 nM. In MDA and HeLa cells there was rather a sustained increase in $\bar{SCPC}$ within the range tested.

To obtain the parametric values of a dose–response relationship (EC50 and SCPCmax) we fitted the data by nonlinear regression to a logistic, dose–response equation of three or four parameters. In all cases, a three parameter equation fitted the data, except in the case of MDA that required a four parameter equation, as shown in the figures and in Table 2. Among the cell lines derived from cervico-uterine cancer, Caski was the one that showed the greatest sensitivity, with an EC50 of 0.6 nM and an SCPCmax of 6; the least sensitive was Hela with an EC50 of 5.8 and an SCPCmax of 5.9. In both breast cancer-derived lines, ouabain induced an increase in SCPC which, while statistically significant, was comparatively smaller compared to that of the other cell lines, with a SCPCmax of only 2.7 and 3.8 for MDA and MCF7, respectively, and an EC50 of 0.002 nM for MCF7 cells and 37.8 nM for MDA cells. The other cell lines had parameters somewhat comparable to that of the cervical cancer-derived lines, with an EC50 of 0.3, 0.6 and 2.0 for SW480, Hpaf-II and A549, respectively, and an SCPCmax of about 5 for all of them.

### 2.1. Octanol Suppresses the Enhancement of GJIC Induced by Ouabain

**Figure 4:**
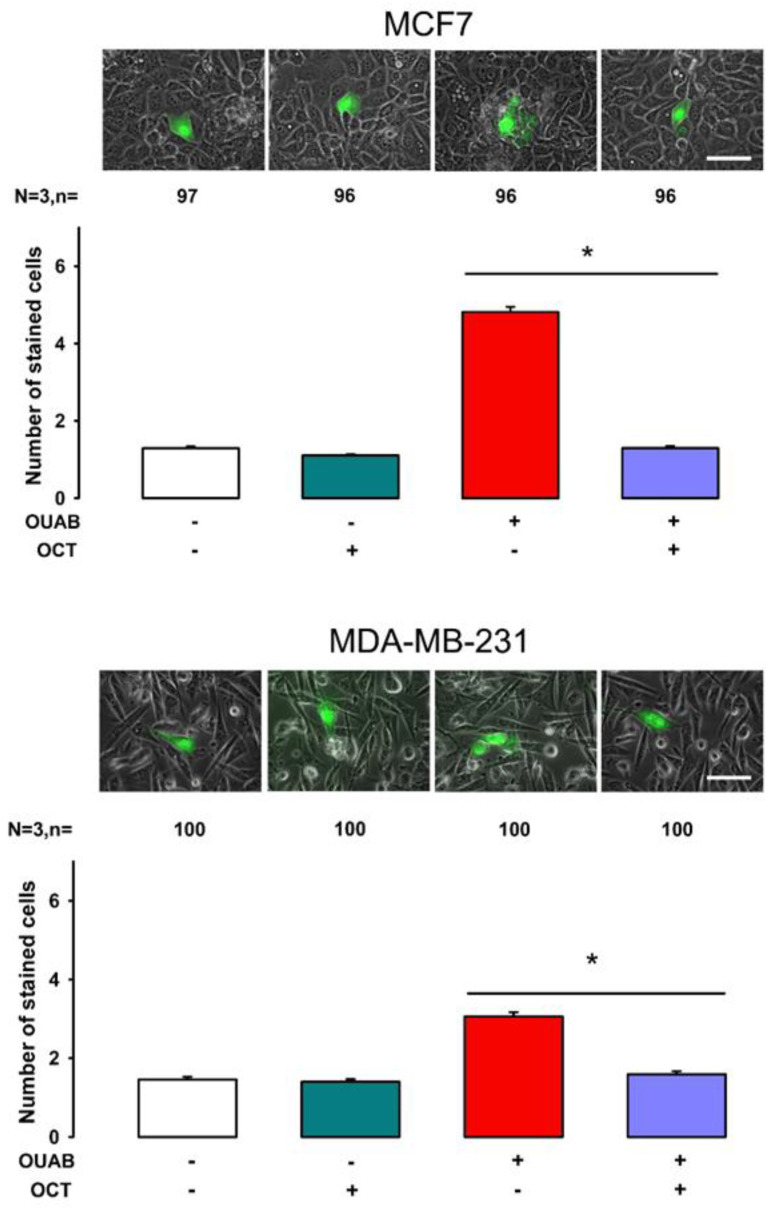
Octanol suppresses the enhancement of GJIC by ouabain. The effect of octanol was assayed in MCF7 (upper) and MDA-MB-231 (lower). The bar chart compares mSCPC (±SE) obtained after three independent trials of dye transfer assays, cells were treated in the conditions indicated at the bottom part of each bar. A representative image of each treatment is shown above the bars. The numbers above each bar indicate the total number of repeats. Asterisks indicate a statistically significant difference (Mann—Whitney Rank Sum Test. * indicates *p* < 0.05). Scale bar = 100 µm.

To prove that neighboring cells get stained through gap junctions, we tested, in MCF7 and MDA-MB-231 cells, the effect of octanol, a widely known gap junction blocker [29,30], on the response observed by ouabain. In both cell types we compared mSCPC with and without treatment of ouabain and octanol. As shown in Figure 4, treatment of MCF7 cells with octanol, without ouabain did not produce a significant change of mSCPC (1.1 ± 0.03, n = 96) compared to that of control condition, (1.2 ± 0.04, n = 97); treatment with ouabain, without octanol produced a significant increase in mSCPC (4.8 ± 0.38, n = 96) compared to control; treatment with ouabain + octanol reduced significantly mSCPC (1.3 ± 0.05) compared to that obtained with ouabain only (Mann—Whitney Rank Sum Test, *p* < 0.001).

Treatment of MDA-MD-231 cells with octanol showed similar results: The mSCPC of cells treated with octanol (1.4 ± 0.07, n = 100) was not significantly different from control (1.46 ± 0.07, n = 100); cell treated with ouabain had a mSCPC (3.06 ± 0.10, n = 100) significantly higher than control (*p* < 0.001); treatment with ouabain + octanol reduced significantly the mSCPC (1.59 ± 0.07, n = 100), compared to that obtained with ouabain only (*p* < 0.001).

These results demonstrate, therefore, that neighboring cells acquire the staining of Lucifer Yellow trough gap junctions.

### 2.2. No Synthesis of New Units of Proteins or mRNA Is Required for Ouabain to Induce the Change of GJIC

**Figure 5:**
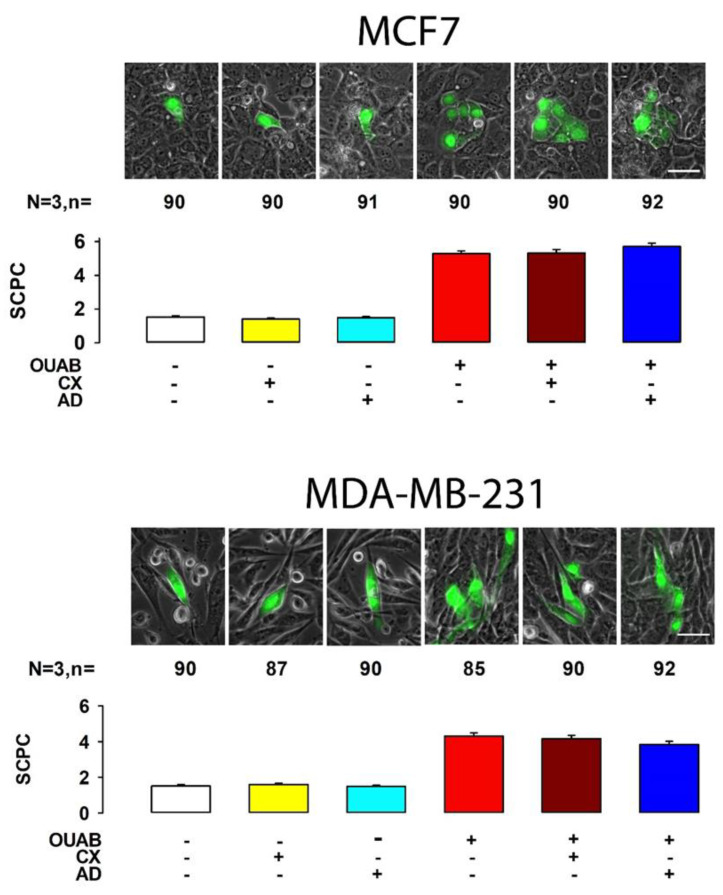
Effect of actinomycin D and cycloheximide on the enhancement of GJIC induced by ouabain in breast cancer cell lines (MCF7 and MDA-MB-231). Each set shown in the upper part represents images of each experimental condition. The lower bar chart compares the mSCPC ± SE of each experimental condition. Scale bar length = 50 µM.

As we described previously, we tested the effect of ouabain on GJIC by varying the concentration of ouabain, but maintaining a constant time of treatment of one hour, this is because in our previous studies with MDCK cells we observed that this is the time at which a maximum response is achieved. In the same previous studies, we showed that during this time no synthesis of new protein units is required to achieve the response. Therefore, in this work we wondered whether cancer derived cell lines require synthesis of RNA, or new protein units for ouabain to induce the increase in GJIC. For this purpose, we probed the effect of cycloheximide, a protein synthesis inhibitor, and actinomycin D, an mRNA synthesis inhibitor, on ouabain-induced GJIC. We conducted three independent dye transfer trials on MDA and MCF7 cells to measure and compare mSCPC under the following conditions: control (untreated), or treated with cycloheximide, actinomycin D, ouabain + cycloheximide, ouabain + actinomycin D. As depicted in Figure 5, MCF7 cells treated with ouabain had a mSCPC of 5.3 ± 0.2 (n = 90) which was statistically different (*p* < 0.001, Mann–Withney) compared to the control (1.5 ± 0.1, n = 90). The mSCPC of cells treated with cycloheximide (1.4 ± 0.1, n = 90) or actinomycin D (1.5 ± 0.1, n = 91) were not statistically different from the control (H = 1.2, *p* = 0.5, Kruskal–Wallis). This indicates that neither drug has an effect per itself. The mSCPC of MCF7 cells treated with ouabain + cycloheximide (5.4 ± 0.2, n = 90) or ouabain + actinomycin D (5.7 ± 0.2, n = 92) were not statistically different (H = 2.1, *p* = 0.34) from that of ouabain (5.3 ± 0.2, n = 90), indicating that neither synthesis of new RNA, nor proteins units are required for ouabain to enhance GJIC. The results with MDA cells were similar, as the mSCPC of cells treated with ouabain (4.3 ± 0.2, n = 90) was statistically different (*p* < 0.001, Mann–Withney) to the control (1.5 ± 0.1, n = 90). The mSCPC of cells treated with cycloheximide (1.7 ± 0.1, n = 86) or actinomycin D (1.6 ± 0.1, n = 90) were not statistically different from the control (H = 1.4, *p* = 0.5, Kruskal–Wallis). The mSCPC values of MDA cells treated with ouabain + cycloheximide (5.4 ± 0.2) or ouabain + actinomycin D (5.7 ± 0.2) were not statistically different that of ouabain (H = 3.1, *p* = 0.21) indicating that neither synthesis of new RNA, nor protein subunits are required for ouabain to enhance GJIC. Therefore, these results indicate that under the experimental conditions used, synthesis of new units of RNA or proteins is not required for ouabain to enhance GJIC in MDA, nor in MCF7 cells.

### 2.3. Involvement of c-Src, ERK1/2 and Rho-ROCK in Ouabain-Induced GJIC

It has been described that, in addition to its role as an electrogenic pump, Na/K ATPase also acts as a signal transducer, coupled to a signalosome, that activates several intracellular signaling pathways [31,32], that may include a c-Src and an IP3-Receptor, both of which activate ERK1/2, which in turn activate diverse cellular processes such as cell growth, apoptosis, and cell motility [33,34,35]. It may also include Rho, a family of small GTPases that participate in a wide variety of cellular functions such as vesicular trafficking, the cell cycle, transcriptomal dynamics, cell polarity, as well as in the organization and modulation of cell junctions by the cytoskeleton [36,37,38,39]. ROCK (Rho-associated protein kinase) is a serine-threonine kinase that as an effector of Rho A, has been reported to be involved in the maintenance of Tigh Junction integrity in endothelial cells [40].

We have shown that both c-Src and ERK1/2 participate in the signaling pathways by which the binding of ouabain to the Na/K ATPase enhances GJIC in epithelial cells [27,28]. For this reason, we considered the possibility that c-Src, ERK1/2 and Rho-ROCK are components participating in the signaling pathway by which ouabain produces changes in GJIC in cancer cell lines. To probe this, we conducted the same experimental approach as already described, in order to compare the effect that ouabain has on GJIC by itself, with that obtained when cells are also treated with specific inhibitors of c-Src, ERK1/2 and Rho-ROCK. To test the involvement of c-Src, we analyzed the effect of PP2 (10 µM for 1 h), a compound that has been shown to be a potent and highly selective Src family tyrosine kinase inhibitor [41,42,43]. To test the participation of ERK1/2, we assayed the effect of PD98059 (25 μM, 1 h), a compound that has been demonstrated to be a potent (IC50 = 4 μM), highly selective and cell-permeable inhibitor of MEK1 and MEK2 [44,45,46,47,48]. To test the involvement of Rho-ROCK, we analyzed the effect of 1 µM Y-27632 (Y27), a cell-permeable, highly potent and selective inhibitor of ROCK, a Rho-associated protein kinase [49].

**Figure 6:**
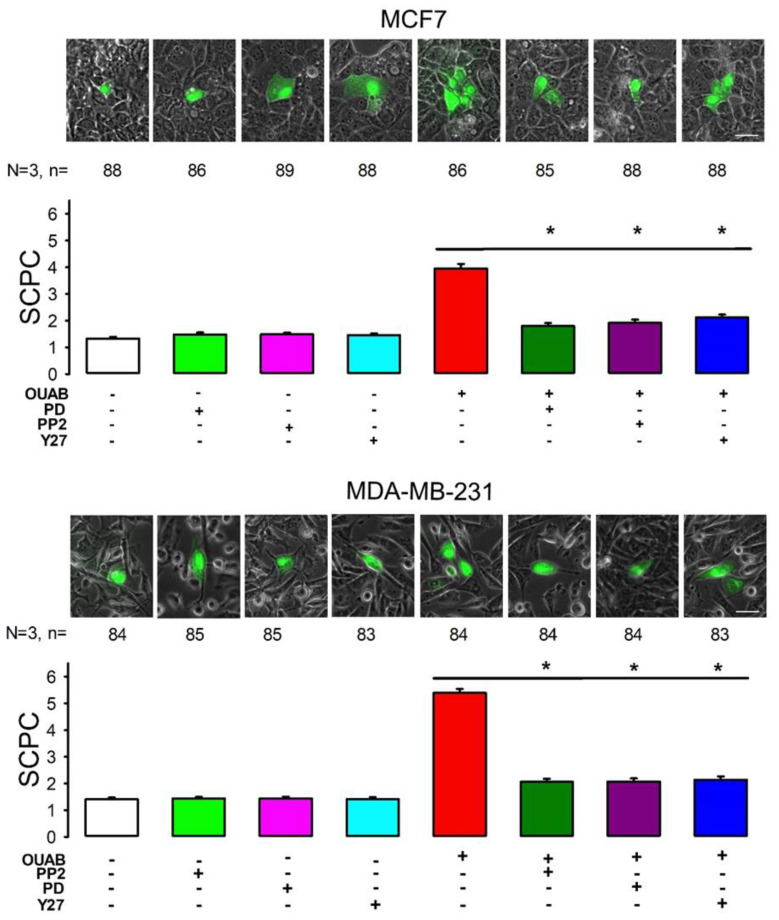
Involvement of c-Src, ERK1/2 and Rho-ROCK on ouabain induced GJIC enhancement. Each set shown, in the upper part, represents images of each experimental condition. The lower bar chart compares mSCPC (±SE) of each experimental condition indicated at the bottom of each bar. Asterisks indicate a statistically significant difference compared to the ouabain group, (Dunn’s method), * indicates *p* < 0.05. Scale bar length = 50 µM.

We assayed the effect of these inhibitors in MCF7 and MDA-MB-231 cells. As shown in Figure 6, the mSCPC after three independent trials were, for MDA: the control (1.3 ± 0.1, n = 88), PD (1.5 ± 0.1, n = 86), PP2 (1.5 ± 0.1, n = 89), Y27 (1.4 ± 0.1, n = 88); ouabain (4.1 ± 0.2, n = 86), ouabain + PD (1.8 ± 0.1, n = 85), ouabain + PP2 (1.9 ± 0.1, n = 88), ouabain + Y27 (2.1 ± 0.1, n = 88). For MCF7: the control (1.4 ± 0.1, n = 84), PD (1.4 ± 0.1, n = 85), PP2 (1.4 ± 0.1, n = 84), Y27 (1.4 ± 0.1, n = 83), ouabain (5.4 ± 0.2, n = 84), ouabain + PD (2.1 ± 0.1, n = 83), ouabain + PP2 (2.1 ± 0.1, n = 84), ouabain + Y27 (2.2 ± 0.1, n = 83).

In both cell lines, inhibitors themselves did not produce any significant effect on GJIC. This is concluded from the fact that, when comparing the groups treated with inhibitors only versus the control, by testing using ANOVA (Kruskal–Wallis), no statistically significant difference was found for MCF7 (H = 0.82, *p* = 0.85), or for MDA (H = 4.0, *p* = 0.25). However, when comparing the group treated only with ouabain with the groups treated with the inhibitors plus ouabain, the Kruskal–Wallis test showed that there is a statistically significant difference for both MCF7 (H = 163, *p* < 0.001) and MDA (H = 114, *p* < 0.001). Subsequently, multiple comparison tests against the control (Dunn’s method) indicate a statistically significant difference (*p* < 0.05) for all inhibitors, on both cell lines. These results indicate that in both cell lines (MCF7 and MDA-MB-231) cSrc, ERK1/2 and Rho-ROCK are included in the signaling mechanisms involved in the effect of ouabain on GJIC.

## 3. Discussion

Cancer is one of the most serious illness worldwide. Despite great medical and scientific advances, there is still a long way to go to reduce the number of people who die in the world as a result of this disease. It is known that cells become cancerous when they lose control of growth and differentiation, which in normal cells is controlled—among other things—by contact inhibition.

Recently it has been found that cardiac glycosides, in addition to their cardiotonic action, influence a wide variety of cellular processes, many of them related to the development and progression of cancerous tumors, such as proliferation, differentiation and motility [1,2,3,4,5,6]. For this reason, some cardiac glycosides, including ouabain, are currently subjects of interest as possible new therapeutic agents to fight cancer.

It is known that there is a relationship between GJIC and cancer, however until now, whether cardiac glycosides influence GJIC in cancer cells has not been explored. For this reason, in this work we tested whether ouabain influences GJIC in a variety of cell lines derived from cancer of cervical origin, breast, lung, colon and pancreas. In each of these cell lines, we evaluated the effect that treatment with ouabain, in concentrations within the nanomolar range (0.1, 1, 10, 50 and 500), produces on GJIC. For each of these concentrations, we injected a certain number of cells with luc ifer yellow, counted the number of cells dyed as a result of injection and statistically compared the average number of cells stained against the control (untreated) group. The fact that the ANOVA (Kruskal–Wallis) test produced significant values (*p* < 0.001) for all selected lines leads us to conclude that ouabain influences GJIC in all these cancer-derived cell lines. Subsequently, multiple comparison tests (Dunn’s method), for each of the ouabain concentrations against the control, indicate that the increase in GJIC begins to be significant from different concentrations, which in most cell lines starts from 1 nM, except for MCF7 cells where this is observed from 0.1 nM. It is interesting to note that in most cell lines, the average number of stained cells increases with concentration to a maximum and then seems to be reduced. In MCF7 cells the average number of stained cells increases at 0.1 nM, then decreases at 1 nM and then increases back to a maximum of 50 nM, followed by a noticeable reduction to 500 nM. Additionally, the behavior of MDA cells is remarkable; the response progressively increased with the dose, without reaching a maximum or a subsequent decrease. Additionally, the EC50 value—calculated from a data regression analysis to fit a dose–response logistic curve, was variable, with the smallest values noted for MCF7 cells (2 pM) and the largest for MDA (37 nM).

Our results are remarkably interesting, considering the background that relates gap junctions, GJIC and cancer: since the first observations made by Loewestein and Kanno more than 60 years ago [15,16], the hypothesis of a relationship between GJIC and cancer has been strengthened with a considerable amount of evidence. On the one hand, an inverse relationship has been shown, i.e., that lack of communication or poor expression of conexins is related to the manifestation of carcinogenic characteristics, but on the other hand, evidence has also been described that shows a direct relationship, i.e., that intense GJIC or overexpression of conexins promotes the development of tumors and even helps migrating tumor cells to invade the surrounding tissue and intra or extravasate. It would appear that the stage of tumor development determines the role that gap junction play [21]. In support of an inverse relationship, it has been described, for instance that: (1) cancer cells, derived from several distinct tissues, notoriously decrease GJIC, as compared to normal tissue [50,51,52,53]; (2) chemical agents that block GJIC also have carcinogenic properties [54]; (3) experimental suppression of connexin expression makes cells more susceptible to becoming carcinogenic [55,56,57]; and (4) transfection of heterologous connexins in cancer cells causes normalization of cell growth control and reduced tumor growth [19,58]. While it is strongly evident that a relationship between GJIC and cancer exists, it is unclear whether this is due to the lack of communication itself or the reduced expression of the connexins that produce it. There are diverse studies showing that it is the lack of GJIC that leads to cancer [15,16,59,60]. There is also a remarkable amount of evidence showing an association between the downregulation of connexins and carcinogenesis [19]. Additionally, there are studies suggesting that connexins have a role as tumor suppressors, regardless of their role as subunits of connexons [61,62]. While the vast majority of the evidence indicates an indirect relationship between gap junctions and cancer, several cases, reported more recently, indicate that intense GJIC or overexpression of connexins in some tumors—or at later tumor stages—encourages tumor growth and their malignity [18,21,22,23]. Some studies indicate that the diapedesis/extravasation process may depend on communication between cancer and endothelial cells [63,64,65].

GJIC is due to expression of gap junction channels or connexons, which in turn consist of six connexins (Cx). There are 21 known connexin isoforms in the human genome, which are named according to their molecular weight (in kD). Most cells and tissues express several connexin isoforms [65,66].

It has been described that lung tissue expresses Cx26, Cx32, Cx37, Cx40, Cx43, and Cx45 [67]; breast tissue: Cx26, Cx32, and Cx43 [68,69]; cervical tissue expresses Cx26, Cx30, Cx40, and Cx43 [70] and prostate tissue has been shown to express Cx26, Cx32, and Cx43 [71,72]. It is worthy to note that, within this variety of expressed connexins, Cx43 appears almost invariably. It is also interesting that when cells become malignant, there is a marked reduction in the expression of various connexins, among them, again connexin 43 stands out (Cx43) [73,74]. Therefore, it is likely that Cx43 plays a special role in the context of the relationship between gap junctions, GJIC and cancer. Because in this work we use a purely functional approach, we cannot determine the types of connexins involved in the increase in ouabain-induced GJIC, or even if the mechanisms involved are common to all types of cancer cells tested here but, in view of the similarity of the results described here with those found in MDCK epithelial cells, in which we showed that the Cx43 is a main player [28], it is possible that Cx43 could be involved in the response obtained from some of the cell types tested here.

Another important fact to highlight is that ouabain was able to induce GJIC in all cell lines, regardless of their phenotype. As already described, in this study we included several cell lines derived from different types of cancer that, although classified as epithelial, differ markedly in their morphological and functional properties, among them MDA-MB-231 stands out. These cells, which have a mesenchymal-like phenotype, have lost their epithelial phenotype, instead exhibiting an elongated spindle-like shape, casting intertwining cellular processes. In addition to their obvious morphology, it has been reported that MDA-MB-231 cells do not express E-cadherin, one of the most representative proteins of epithelial cells [75]. In addition, component proteins of tight junctions, such as occludins and claudins are downregulated or silenced [76]. On the other hand, studies suggest that, in epithelial cells, gap junctions operate in a dependent and coordinated manner on the other cell–cell contact structures (tight junctions, adherens junctions and demosomes [77,78,79,80,81,82,83].

For this reason, we assumed that the MDA-MB-231, would not respond by increasing GJIC in response to treatment with ouabain but, as shown, they did. This leads us to believe that the expression of an epithelial phenotype is not an indispensable requirement for ouabain to exert its inducing effect on GJIC.

The fact that no synthesis of mRNA nor proteins is required, leads us to suggest that ouabain produces this effect through the relocation of subunits of previously synthesized connexins, or to a change in the opening kinetics of the gap junctions already assembled in the membrane. On the other hand, the change in GJIC induced by ouabain, although statistically significant was not very conspicuous. This suggests that ouabain is not a decisive factor influencing GJIC, but rather it has a contribution that may have to be synergized by other molecular mechanisms still not characterized. It should also be noted that in these studies we used a time range of only one hour, in which, as we describe, we found that no synthesis of mRNA or proteins is required, but that does not exclude the possibility that, with longer treatment times, ouabain will operate other mechanisms that induce protein synthesis and therefore could produce a more conspicuous response.

Finally, we must realize that, while in this work we have shown that ouabain induces an increase in GJIC, this does not necessarily imply that it is one of the mechanisms involved in therapeutic action against cancer, although it is a precedent worth considering to address subsequent studies.

## 4. Materials and Methods

### 4.1. Cell Culture

CaSki is an epithelial cell line derived from epidermoid carcinoma of the cervix metastatic to the small bowel mesentery. It was obtained from a 40 year old Caucasian female who had previously undergone irradiation and surgical treatment of the malignancy [84]. CaSKi cells were obtained from ATCC (CRL-1550) and cultured in RPMI1640 medium, supplemented with penicillin-streptomycin 10,000 U/µg/mL (Cat. 15140122, Thermo Fisher Scientific, Waltham, MA, USA) and 10% GIBCO FBS (Thermo Fisher, A4766801).

SiHa is an epithelial, adherent cell line, derived from an Asian, 55 year old female who suffered from human papillomavirus-related cervical squamous cell carcinoma [85]. SiHa cells were obtained from ATCC (HTB-35) and cultured in DMEM medium, supplemented with penicillin–streptomycin 10,000 U/µg/mL and 10% GIBCO FBS. Hela cells were obtained from Dr. Camacho (Department of Pharmacology, CINVESTAV, Mexico City, Mexico).

HeLa is an epithelial, adherent cell line, derived from a cervical adenocarcinoma, taken from a black, 31 year old female. It was the first human cell line to prove successful in vitro [86]. Hela cells were cultured in DMEM medium, supplemented with penicillin-streptomycin 10,000 U/µg/mL and 10% GIBCO FBS.

MDA-MB-231 is an adherent, epithelial cell line derived from a pleural effusion of a 51 year old Caucasian female with a metastatic mammary adenocarcinoma. It is a highly aggressive, invasive and poorly differentiated triple-negative breast cancer (TNBC) cell line, as it lacks oestrogen receptor (ER) and progesterone receptor (PR) expression, as well as HER2 (human epidermal growth factor receptor 2) amplification [87]. MDA-MB-231 were obtained from ATCC (HTB 26) and cultured in RPMI 1640 media supplemented with 10,000 U/μg/mL penicillin-streptomycin and 10% FBS.

MCF7 is an adherent cell line with characteristics of differentiated mammary epithelium, derived from an invasive breast ductal carcinoma, taken from a Caucasian, 67 year old female [88]. MCF-7 cells were obtained from ATCC (HTB-22) and cultured in DMEM F-12 media, supplemented with 10,000 U/μg/mL penicillin–streptomycin and 10% FBS.

A549 is an adherent, epithelial cell line, derived from explant of a biopsy of lung carcinoma of a 58 year old caucasian male [89]. A549 cell line was kindly donated by Dr. Javier Camacho (Department of Pharmacology, CINVESTAV). A549 cells were cultured in F12K medium, supplemented with penicillin–streptomycin 10,000 U/µg/mL and 10% GIBCO FBS.

SW480 is an adherent, epithelial cell line, isolated from a primary adenocarcinoma arising in the colon [90]. The SW480 cell line was kindly donated by Dr. Camacho (Department of Pharmacology, CINVESTAV). A549 cells were cultured in DMEM/F12 medium (Invitrogen, 12400024, Thermo Fisher Scientific, Mexico City, Mexico), supplemented with penicillin-streptomycin 10,000 U/µg/mL and 10% FBS.

HPAF-II is an adherent, epithelial cell line, derived from the ascitic fluid of a male, 44 year old, Caucasian patient with pancreatic adenocarcinoma [91,92]. The HPAF-II cell line was kindly donated by Dr. Jose Segovia (Department of Physiology, CINVESTAV). HPAF-II cells were cultured in RPMI1640 medium, supplemented with penicillin–streptomycin 10,000 U/µg/mL and 10% FBS. All cell lines were cultured in a 5% CO_2_ atmosphere at 36.5 °C.

For dye transfer assays, cells were seeded at 80% confluence on sterile glass coverslips, deposited on 24-well multiwall plates (Corning, Costar plates-Merck, Darmstadt, Germany). Once confluence was reached, cells were depleted, by reducing FBS to 1% during 24 h before ouabain treatments.

### 4.2. Measurement of Gap Junctional Intercellular Communication by Dye Transfer Assays

Cells to be tested were seeded at 80% confluence on glass coverslips and incubated with a medium containing 10% Fetal Bovine Serum. After 24 h the FBS in the culturing media was reduced to 1%, to avoid the possibility of contaminating ouabain, and the cells were kept in this, depleted media, for 24 h before treatments. Subsequently, the coverslips were incubated for one hour with depleted media plus ouabain at different concentrations in the nanomolar range (0, 0.1, 1, 10, 50 and 500 nM). Micropipettes were elaborated from borosilicate glass capillaries tubes (Kimax, 34500-99) on a vertical David-Kopf puller (DKI-700c). Those with a tip electrical resistance of 5–10 MOhms were backfilled with a saline solution containing 120 mM KCl, 5 mM NaCl, 1 mM MgCl2, and 5 mM HEPES, (pH 7.4) and Lucifer Yellow (1%). After filling up, pipettes were attached to holder device, which was mounted to a micromanipulator (PCS-750; Burleigh Instruments, NY, USA). Coverslips on which cell monolayers had been grown, were placed in a translucent chamber, filled with PBS plus Ca^2+^ (1.8 mM) solution, at room temperature. For impalement of cells the chamber was mounted on the stage of an inverted microscope (Diaphot 300; Nikon, Tokyo, Japan) equipped with epifluorescence. Three independent trials were made on each cell line. On each trial, about 30 repeats were made per coverslip. In each repeat, cells were randomly chosen, from among those constituting the monolayer, then impaled and injected, one at a time, using a pneumatically driven microinjecting device (IM300; Narishige, NY, USA). After about 30 to 50 cells injected, the coverslips were rinsed with PBS and fixed by dipping into 4% paraformaldehyde, then rinsed (3×) with PBS and mounted using VECTASHIELD^®^ (H-1000; Vector Laboratories, Burlingame CA, USA). Eight-bit images of the fluorescent cells were acquired at room temperature using a Zeiss M200 inverted microscope equipped with a Plan-NeoFluar 63 × N.A. 1.25 objective lens, an AxioCam MRm camera and Axovision 4.8 software (www.axovision.com). The captured images were imported into FIJI Is Just ImageJ software (release 2.8, NIH, Bethesda, MD, USA) to adjust the brightness and the contrast and GIMP (release 2.8.10, NIH) to compose the figures.

### 4.3. Chemicals

Ouabain (O-3125; Sigma-Aldrich, St. Louis, MO, USA) was prepared in DMSO. Lucifer Yellow was obtained from Sigma-Aldrich (67764-47-5). In the corresponding assays cells were exposed to 10 μM PP2, an inhibitor of c-Src kinase (MEK-1; 513000; Merck Millipore, Darmstadt, Germany) and 25 μM PD98059, an inhibitor of mitogen extracellular kinase-1 (529573; Merck Millipore). Y-27632 (Merck KGaA, Cat 688000, Darmstadt, Germany), was prepared as a 10 mM stock in water, and used at a concentration of 1 µM.

### 4.4. Statistical Analyses

The data collected in this work was processed and analyzed statistically using the Microsoft Office 365 Excel application and Sigmaplot 12.5. The data were generated by counting the stained cells of each cluster, the product of the injection of a single cell. The results shown are the product of three independent experimental trials. The number of data points is indicated in the figures and in the text. The data are represented as the average values and dispersion as the standard error of the mean (SE). Statistical analysis, as indicated in the text and figures, was contained in a ONEWAY test, followed by multiple comparison tests, in the event that the data did not meet the normality criterion required for a parametric analysis, the Kruskal–Wallis non-parametric test was used, followed by a paired comparison test with respect to a control (Dunn’s method). A minimum criterion of *p* < 0.01 was considered for a statistically significant difference.

## Figures and Tables

**Table 1:** Statistical analysis of the effect of ouabain on cancer lines. The columns below the pink panel show, for each concentration tested: The mSCPC, standard error, number of repeats and the result of a paired comparison vs. control (*p* < 0.05). The green part shows the result of an ANOVA (Kruskal–Wallis) test, the H value, degrees of freedom, and whether or not it is statistically significant (*p* < 0.001).

Cell Line	Ouabain (nM)																							ANOVA		
	0			0.1				1				10				50				500						
	mSCPC	SE	n	mSCPC	SE	n	*p* < 0.05	mSCPC	SE	n	*p* < 0.05	mSCPC	SE	n	*p* < 0.05	mSCPC	SE	n	*p* < 0.05	mSCPC	SE	n	*p* < 0.05	H	d.f.	*p* < 0.001
CaSki	1.4	0.1	113	2.0	0.1	113	No	2.7	0.1	126	Yes	6.5	0.2	128	Yes	5.9	0.2	127	Yes	5.1	0.1	126	Yes	472.0	5	Yes
SiHa	1.3	0.1	98	1.5	0.1	99	No	2.1	0.1	99	Yes	2.7	0.1	99	Yes	5.3	0.2	98	Yes	4.3	0.1	99	Yes	407.6	5	Yes
HeLa	1.1	0.0	68	1.2	0.1	67	No	2.0	0.1	67	Yes	2.9	0.2	69	Yes	4.4	0.2	68	Yes	4.8	0.2	68	Yes	274.5	5	Yes
MDA	1.7	0.1	231	1.8	0.1	267	No	2.0	0.1	256	Yes	2.1	0.1	260	Yes	2.6	0.1	278	Yes	3.0	0.1	215	Yes	141.1	5	Yes
MCF7	2.0	0.1	147	3.3	0.1	137	Yes	2.8	0.1	120	Yes	4.3	0.1	138	Yes	4.8	0.2	71	Yes	2.7	0.1	78	Yes	208.3	5	Yes
A549	1.4	0.1	94	1.6	0.1	95	No	1.6	0.1	92	No	3.5	0.1	96	Yes	5.0	0.1	97	Yes	4.6	0.1	97	Yes	397.9	5	Yes
SW480	1.7	0.1	95	1.9	0.1	95	No	3.3	0.1	95	Yes	4.6	0.1	95	Yes	5.9	0.2	96	Yes	4.5	0.1	96	Yes	376.7	5	Yes
Hpaf1	1.1	0.1	67	1.2	0.1	65	No	2.5	0.2	66	Yes	5.4	0.3	67	Yes	5.2	0.2	67	Yes	4.6	0.2	68	Yes	274.0	5	Yes

**Table 2:** Parametric values resulting after adjusting the experimental data, by regression, to the dose–response curve, logistics of three or four parameters, the last column shows the value of the correlation coefficient.

Cell Line	SCPCmax	Hill Coef	EC50	S0	R
CaSki	6.0	−0.7	0.6		0.6
SiHa	5.3	−0.4	2.1		0.7
HeLa	5.9	−0.4	5.8		0.8
MDA	2.7	−1.0	37.8	1.9	0.5
MCF7	3.8	−0.4	2.1 × 10^−3^		0.3
A549	5.3	−0.5	2.0		0.8
HPAF1	5.1	−0.8	0.6		0.7
SW480	5.7	−0.6	0.3		0.7
